# 5-HT_7_ Receptors Regulate Excitatory-Inhibitory Balance in Mouse Spinal Cord Dorsal Horn

**DOI:** 10.3389/fnmol.2022.946159

**Published:** 2022-07-06

**Authors:** Antonella Comitato, Enza Lacivita, Marcello Leopoldo, Rita Bardoni

**Affiliations:** ^1^Department of Biomedical, Metabolic and Neural Sciences, University of Modena and Reggio Emilia, Modena, Italy; ^2^Department of Pharmacy – Drug Sciences, University of Bari Aldo Moro, Bari, Italy

**Keywords:** pain, serotonin, spinal cord, synaptic transmission, GABA, electrophysiology

## Abstract

Serotonergic receptors of the 5-HT_7_ type (5-HT_7_Rs) are widely expressed in the central nervous system (CNS), where they modulate several functions, such as pain. Behavioral experiments *in vivo* have shown both anti- and pro-nociceptive actions of 5-HT_7_Rs, although an analgesic effect seems to be prevalent. In the spinal cord dorsal horn, the mechanisms involved in 5-HT_7_R-mediated synaptic modulation are still poorly understood, especially those regarding the control of synaptic inhibition. The present study investigated the modulation exerted by 5-HT_7_Rs on dorsal horn excitatory and inhibitory synaptic circuits, by performing patch-clamp recordings from lamina II neurons in mouse spinal cord slices. Our results show that applying the selective 5-HT_7_ agonist LP-211 facilitates glutamatergic release by enhancing the frequency of spontaneous postsynaptic currents (sEPSCs) and increasing the peak amplitude of excitatory postsynaptic currents (EPSCs) evoked by dorsal root stimulation. The effects on sEPSCs were still observed in the presence of the 5-HT_1*A*_ antagonist WAY-100635, while the 5-HT_7_ antagonist SB-269970 blocked them. LP-211 was also able to increase the release of gamma-aminobutyric acid (GABA) and glycine, as shown by the increase of spontaneous inhibitory currents (sIPSC) frequency and evoked inhibitory postsynaptic currents (IPSC) amplitude. LP-211 was proved to be more effective in potentiating synaptic inhibition as compared to excitation: consistently, 5-HT_7_R activation significantly enhanced the excitability of tonic firing neurons, mainly corresponding to inhibitory interneurons. Our data bring new insights into the mechanisms of synaptic modulation mediated by 5-HT_7_Rs in the dorsal horn. Stronger impact on synaptic inhibition supports the hypothesis that these receptors may play an anti-nociceptive role in the spinal cord of naïve animals.

## Introduction

Serotonergic fibers modulate pain transmission in the spinal cord dorsal horn through the activation of several serotonergic receptors (5-HTRs). Among these, the most recently identified 5-HT receptor family, the 5-HT_7_ receptor (5-HT_7_R), has been involved in pain modulation, acting at several levels along the pain axis (Cortes-Altamirano et al., 2018; Bardoni, 2019). 5HT_7_Rs are heterodimeric receptors that stimulate cyclic adenosine monophosphate (cAMP) formation by activating adenylate cyclase through a stimulatory Gs protein, which also causes the activation of RAS-dependent extracellular-regulated kinases (ERKs) (Norum et al., 2003). Targets of 5-HT_7_R-mediated modulation are voltage-dependent ion channels, membrane transporters, and synaptic receptors. Indeed, 5-HT_7_Rs have been reported to induce neuronal depolarization, regulate neurotransmitter release, and modulate synaptic plasticity in several areas of the central nervous system (CNS) (Ciranna and Catania, 2014).

In the nociceptive system, expression of 5-HT_7_Rs has been detected in dorsal root ganglion cells (DRGs), especially on small- and medium-size cell bodies and in the dorsal horn, on both axons and cell bodies (Meuser et al., 2002; Doly et al., 2005). Labeling for 5-HT_7_Rs in the spinal cord appeared to be particularly intense in the superficial dorsal horn, compatibly with a role of these receptors in pain transmission.

The effects of 5-HT_7_Rs on pain have been investigated *in vivo* in different animal models: although an anti-nociceptive action seemed to be prevalent in both inflammatory and neuropathic pain, pro-nociceptive effects have also been reported (reviewed in Cortes-Altamirano et al., 2018; Bardoni, 2019). These discrepancies may depend on the site of 5-HT_7_R activation (periphery vs. CNS), the use of different pain models and animal species, and the scarce availability of selective 5-HT_7_R agonists. Recently, more selective agonists for 5-HT_7_R have become available (Blattner et al., 2019). Among these, the compound LP-211 has been tested in several studies, proving to be specific and effective in activating 5HT_7_Rs, both *in vivo* and *in vitro* (Leopoldo et al., 2011; Costa et al., 2012; Demirkaya et al., 2016; Lippiello et al., 2016).

Data about the modulatory effects exerted by 5-HT_7_Rs on nociceptive neurons are limited. An early study had shown that activation of 5-HT_7_Rs with 5-Carboxamidotryptamine increased the amplitude of Ih current in medium diameter rat DRG neurons (Cardenas et al., 1999). In mouse trigeminal subnucleus caudalis, application of the 5HT_1*A/*7_ agonist 8-hydroxy-2-(di-n-propylamino)tetralin (8-OH-DPAT; in the presence of a 5-HT_1*A*_ antagonist) produced a depolarization in a subpopulation of neurons (Yang et al., 2014), while in rat deep dorsal horn 8-OH-DPAT facilitated excitatory synaptic responses (Garraway and Hochman, 2001).

In the spinal cord superficial dorsal horn, the role of 5-HT_7_Rs in modulating excitatory and inhibitory synaptic circuits has not been clarified. By patch-clamp recording synaptic responses from neurons in mouse spinal cord slices, we have studied the effects of the selective 5-HT_7_ agonist LP-211 on synaptic transmission in the superficial dorsal horn. Our results show that 5-HT_7_Rs can potentiate the release of glutamate and gamma-aminobutyric acid (GABA)/glycine in this region, exerting a more powerful effect on synaptic inhibition and excitability of inhibitory interneurons.

Parts of these results have been presented in a preliminary form (Comitato et al., 2021).

## Materials and Methods

### Spinal Cord Slice Preparation

The Italian Ministry of Health approved all the experiments conducted on postnatal CD1 mice of either sex (P18–P28) following the Guide for the Care and Use of Laboratory Animals and the EU and Italian regulations on animal welfare. Spinal cord slices were obtained following the procedure described previously (Betelli et al., 2015; Bardoni et al., 2019). Briefly, the animals were anesthetized with isoflurane and decapitated; the spinal cord and vertebrae were rapidly removed and placed in ice-cold dissecting Krebs’ solution (composition in mM: 95 NaCl, 50 sucrose, 2.5 KCl, 1.25 NaH_2_PO_4_, 26 NaHCO_3_, 25 glucose, 6 MgCl_2_, 1.5 CaCl_2_, and 1 kynurenic acid, pH 7.4, 320 mOsm), bubbled with 95% O_2_ and 5% CO_2_. The lumbar spinal cord was isolated, embedded in an agarose block (low melting point agarose 3%, Thermo Fisher Scientific, Waltham, MA, United States), and transverse slices (400–500 μm thick) were obtained using a vibrating microtome (WPI, Sarasota, FL, United States). The slices were maintained in oxygenated incubation Krebs’ solution (in mM: 125 NaCl, 2.5 KCl, 1.25 NaH_2_PO_4_, 26 NaHCO_3_, 25 glucose, 6 MgCl_2_, and 1 CaCl_2_, pH 7.4, 320 mOsm) at 35°C for 20 min and then used for recording.

### Patch-Clamp Recording and Stimulation

The patch-clamp recording in whole-cell configuration was performed on lamina II neurons at room temperature. The slices were perfused at 2 ml/min with recording Krebs’ solution (in mM: 125 NaCl, 2.5 KCl, 1.25 NaH_2_PO_4_, 26 NaHCO_3_, 25 glucose, 1 MgCl_2_, and 2 CaCl_2_, pH 7.4, 320 mOsm). Recordings of excitatory postsynaptic currents (EPSCs) were performed in voltage-clamp at −70 mV by using a potassium-based intracellular solution with the following composition (in mM): 120 potassium methanesulfonate, 10 NaCl, 10 ethylene glycol bis(2-aminoethyl ether)tetraacetic acid (EGTA), 1 CaCl_2_, 10 hydroxyethyl piperazineethanesulfonic acid (HEPES), 5 adenosine triphosphate (ATP)-Mg, 0.5 guanosine triphosphate (GTP)-Na, pH adjusted to 7.2 with KOH, and osmolarity 300 mOsm. The data were recorded and acquired using a MultiClamp 700A Amplifier and the pClamp 10 software (Molecular Devices, Sunnyvale, CA, United States). The sampling rate was 10 kHz, and the data were filtered at 2 kHz. Series resistance was not compensated, and cells with a resistance higher than 30 MOhm were discarded. Junction potentials were corrected off-line. Inhibitory postsynaptic currents (IPSCs) were recorded in voltage-clamp at 0 mV by using a cesium-based intracellular solution having the following composition (in mM): 130 cesium methanesulfonate, 10 sodium methanesulfonate, 10 EGTA, 1 CaCl_2_, 10 HEPES, 2 ATP-Mg, pH adjusted to 7.2 with CsOH, and osmolarity 300 mOsm.

Evoked EPSCs were obtained by stimulating the dorsal root attached to the slice with a suction electrode. The applied stimuli (500 μA intensity and 0.1 ms duration) activated both Aδ and C fibers synapsing onto lamina II neurons. Monosynaptic EPSCs were identified from the absence of failures during a train of 20 stimuli at 1 Hz (Daniele and MacDermott, 2009; Bardoni et al., 2019).

Evoked IPSCs were elicited by focally stimulating the region around the recorded cell with a glass pipette (tip diameter 2–5 μm) at an intensity of 0.1–1 mA and duration of 0.1 ms. Monosynaptic IPSCs were recognized by constant latency and by the absence of failures at 1 Hz.

Single EPSCs or IPSCs were recorded by stimulating at 0.1 Hz. EPSCs or IPSCs recorded with the paired-pulse protocol were evoked by applying 2 stimuli at 400 ms intervals. The time between consecutive pairs of stimuli was 20–30 s.

Recordings in the current clamp were performed by using the potassium-based intracellular solution. Resting potential was determined within the first 5 min of recording: cells with membrane potential more positive than −50 mV were discarded. The neuronal firing pattern was determined by applying at least 10 current steps (amplitude: 10–20 pA; duration: 500 ms). Between the steps, the membrane potential was held at about −80 mV to unmask the delayed firing pattern better, as previously described (Yasaka et al., 2010; Bardoni et al., 2019). Once the firing pattern was established, root stimulation was performed as described above, using the paired-pulse protocol to record paired excitatory postsynaptic potentials (EPSPs). The neuron potential was maintained at −60/−65 mV.

### Data Analysis

**FIGURE 1:**
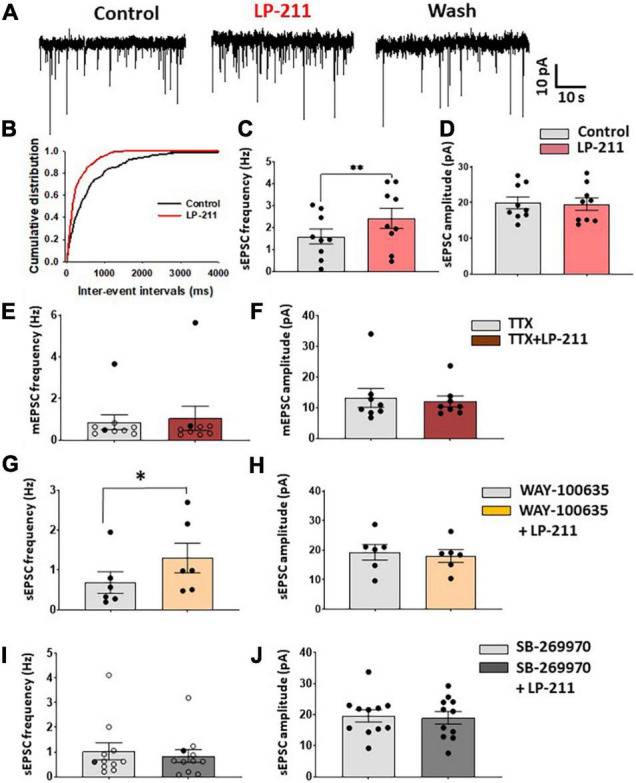
Application of LP 211 increases spontaneous EPSCs in a subpopulation of lamina II neurons. **(A)** Examples of spontaneous EPSC recordings (sEPSCs), obtained in voltage-clamp at –70 mV. LP-211 (1 μM) caused a significant increase in sEPSC frequency, which was reversible in the wash. **(B)** Cumulative distributions of the inter-event intervals determined from the neuron shown in panel **(A)**. LP-211 caused a shift of the curve to the left, indicating an increase in sEPSC frequency (Kolmogorov–Smirnov test, *p* = 0.000). **(C)** Summary graph of sEPSC frequencies obtained from the neurons responsive to LP-211 (9 out 14 tested cells; paired *t*-test, *p* < 0.01); **(D)** LP-211 did not change sEPSC amplitude in the responsive cells (paired *t*-test, *p* = 0.69, *n* = 9). **(E)** Tetrodotoxin (TTX, 1 μM) reduced the effect of LP-211 on spontaneous glutamate release: in only 2 neurons (black circles) out of 9 a significant increase of mEPSC frequency was observed. The overall effect of LP-211 on mEPSC frequency was not significant (Wilcoxon rank-sum test, *p* = 0.91). **(F)** No changes in mEPSC amplitude were observed in LP-211 + TTX (Wilcoxon rank-sum test, *p* = 0.96, *n* = 9). **(G,H)** Co-application of the 5-HT_1*A*_ antagonist WAY 100-635 (10 μM) did not prevent the increase of sEPSC on 6 out of 10 tested neurons (paired *t*-test on responsive cells, *p* < 0.05, *n* = 6). The sEPSC amplitude was not significantly changed (paired *t*-test, *p* = 0.39, *n* = 6). **(I)** Co-application of LP-211 with the 5-HT_7_ antagonist SB-269970 (10 μM) blocked both the LP-211 effects on sEPSC frequency [**(I)**: only one responsive cell (black circles) out of 11 tested, Wilcoxon rank-sum test, *p* = 0.17, n = 11] and amplitude [**(J)**: paired *t*-test, *p* = 0.4, *n* = 11]. Asterisks reported in the graphs represent statistical significance: *p < 0.05; ^**^p < 0.01; ^***^p < 0.001.

Spontaneous EPSCs or IPSCs were analyzed offline using pClamp10 software and Minianalysis (Synaptosoft, United States). The responsivity of individual cells to LP-211 was assessed by performing the Kolmogorov–Smirnov test on cumulative distributions of inter-event intervals (Figure 1B). Evoked EPSCs and IPSCs were analyzed using pClamp10 software: sensitivity to LP-211 was established by comparing with an unpaired *t*-test, the peak amplitudes of 5–10 currents in control and in the presence of the 5-HT_7_ agonist. Paired pulse ratio was determined as the ratio between the second and the first EPSC.

The data are expressed as the mean ± SEM, and differences were considered significant for *p* < 0.05. Comparisons between 2 groups were performed by using an unpaired or paired t-test. Non-parametric tests were applied when the data were not normally distributed. Graphs and statistical analysis were obtained by using GraphPad Prism 9.3 (GraphPad Software, San Diego, CA, United States).

### Drugs

All the components of Krebs and intracellular solutions were obtained from Sigma-Aldrich (Merck Group, Darmstadt, Germany). LP-211 tartrate was provided by Prof. Marcello Leopoldo at the University of Bari (Italy). Aliquots of 1 μM stock solution in dimethyl sulfoxide (DMSO) were initially prepared and then diluted to 1 μM in recording Krebs’ solution on the day of the experiment. NBQX, D-AP5, and tetrodotoxin (TTX) were provided by Abcam (Cambridge, United Kingdom), and WAY-100635 and SB-269970 were obtained from Sigma-Aldrich.

## Results

We have tested the effects of 5-HT_7_Rs on synaptic transmission in the superficial dorsal horn by recording from 109 lamina II neurons in mouse spinal cord slices. To activate 5-HT_7_Rs, we have bath-applied the selective agonist LP-211 at 1 μM. Application of LP-211 to the slice for 3–5 min caused a significant increase of glutamate release in a subpopulation of lamina II neurons, recorded in voltage-clamp at −70 mV (Figures 1A–C). The responsivity to LP-211 was assessed on individual cells based on the significant change in frequency of spontaneous postsynaptic currents (sEPSCs; Figure 1B). The 5-HT_7_R agonist caused a significant increase in sEPSC frequency in a subpopulation of neurons (mean percentage increase: 86.9 ± 32.9%, n = 9 out of 14), which was reversible in the wash and it was not accompanied by a change in sEPSC amplitude (Figure 1D). In some neurons, we also observed a slow inward current during LP-211 application (mean amplitude: 8.9 ± 1.1 pA; mean duration: 3.5 ± 0.3 min; 5 out of 11 neurons), indicating the activation of postsynaptic 5-HT_7_Rs located on the recorded cell.

LP-211 had been shown to be effective in activating 5-HT_7_Rs in brain slices at concentrations in the nanomolar range (Costa et al., 2012), so we tested the agonist at 0.1 μM on a sample of 8 neurons. We observed a significant increase in sEPSC frequency only in one neuron, and the analysis of the whole sample did not show a significant effect of 0.1 μM LP-211 (mean frequency in control: 0.58 ± 0.13 Hz; LP-211: 0.57 ± 0.13 Hz; paired t-test *p* = 0.93, *n* = 8).

To test whether the effect of LP-211 on glutamate release was dependent on action potential firing, we applied 1 μM LP-211 in the presence of 1 μM tetrodotoxin (TTX). In these conditions, LP-211 significantly increased miniature EPSC (mEPSC) frequency in only 2 out of 9 tested lamina II neurons, while the mEPSC amplitude was unaffected (Figures 1E,F). This would suggest that the potentiating effect exerted by 5-HT_7_Rs partially involves the generation of action potentials in the excitatory networks. Furthermore, the lack of effect on mEPSC amplitude indicates that 5-HT_7_Rs act at the presynaptic site.

Since it is known that some 5-HT_7_ agonists can also activate 5-HT_1*A*_Rs, we have tested 1 μM LP-211 in the presence of the 5-HT_1*A*_ antagonist WAY-100635 (10 μM) to exclude the involvement of these receptors in the modulation of glutamate release. As shown in Figures 1G,H, a significant increase in sEPSC frequency is still observed in 6 out of 10 tested cells, without any significant alteration in sEPSC amplitude. Conversely, the LP-211 effect on sEPSCs was blocked by applying the 5-HT_7_ antagonist SB-269970, confirming the selective activation of 5-HT_7_Rs by the agonist (Figures 1I,J).

**FIGURE 2:**
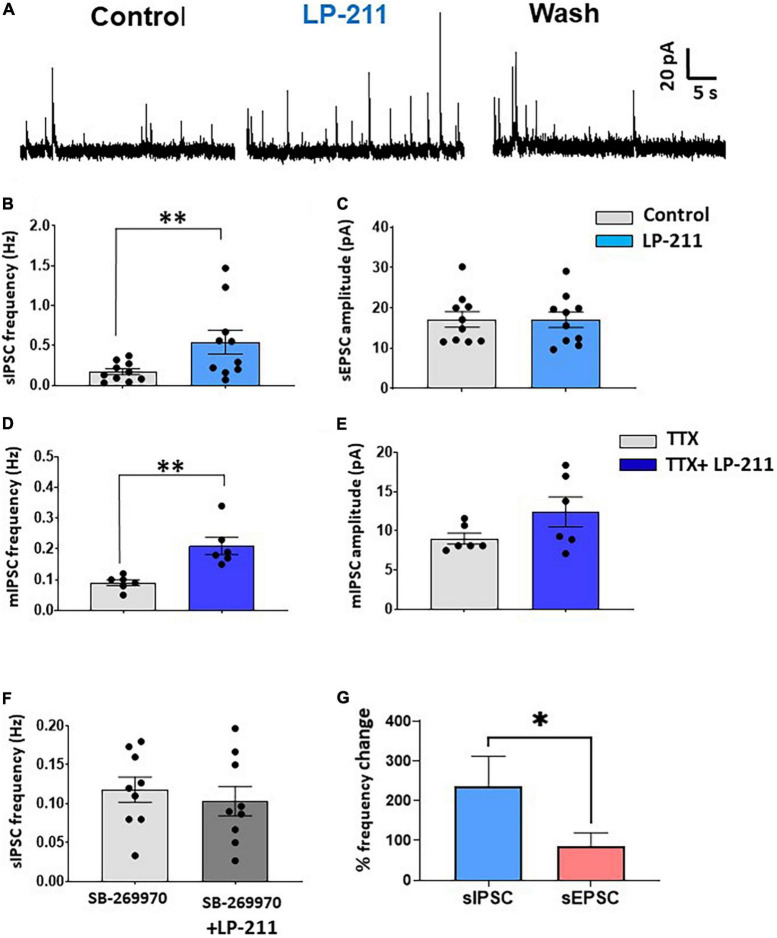
Activation of 5-HT_7_Rs by LP-211 potentiates spontaneous GABA and glycine release. **(A)** Representative traces of spontaneous inhibitory postsynaptic currents (IPSCs), recorded in voltage-clamp at 0 mV, in the presence of the AMPA and NMDA receptor antagonists NBQX (10 μM) and D-AP5 (50 μM). An increase in sIPSC frequency was evident in the presence of LP-211 (1 μM). **(B,C)** Summary graphs obtained from the lamina II neurons responsive to LP-211. The agonist caused a significant increase of sIPSC frequency in 10 out of 14 tested cells [**(B)** paired *t*-test, *p* < 0.01, *n* = 10], without affecting the mean sIPSC amplitude (paired *t*-test, *p* = 0.96, n = 10). **(D,E)** In the presence of 1 μM TTX, LP-211 still produced a significant increase in mIPSC frequency in 6 out of 11 cells tested [**(D)** paired t-test, *p* < 0.01, *n* = 6], while mean mIPSC was not altered [**(E)** paired *t*-test, *p* = 0.12, *n* = 6]. **(F)** Co-application of LP-211 with SB-269970 (10 μM) inhibited the increase of sEPSC frequency, confirming the involvement of 5-HT_7_Rs (zero responsive cells out of 9 tested, paired *t*-test, *p* = 0.21, *n* = 9). **(G)** LP-211 exerted a stronger effect on sIPSC frequency as compared to sEPSC (Mann–Whitney test, *p* < 0.05, *n* = 10 and 9, for sIPSCs and sEPSCs, respectively). Asterisks reported in the graphs represent statistical significance: *p < 0.05; ^**^p < 0.01; ^***^p < 0.001.

Activation of 5-HT_7_Rs by LP-211 was also effective in potentiating spontaneous inhibitory currents (sIPSCs) mediated by GABA and/or glycine and recorded in voltage-clamp at 0 mV (Figure 2). In the presence of Alpha-Amino-3-Hydroxy-5-Methyl-4-Isoxazole Propionic Acid (AMPA) and N-methyl-D-aspartate (NMDA) receptor antagonists NBQX and D-AP5 (10 and 50 μM, respectively), 1 μM of LP-211 strongly increased sIPSC frequency in 10 out of 14 lamina II neurons (Figures 2A,B; mean percentage increase: 236.7 ± 75.2%), without changing sIPSC amplitude (Figure 2C). In the presence of TTX, a significant enhancement of mIPSC frequency was still observed in 6 out of 11 neurons (Figure 2D). Although the overall mIPSC amplitude of the responsive cells was not significantly changed by LP-211 (Figure 2E), the increase in frequency was accompanied by an enhancement of amplitude in three cells. The analysis of the frequency distributions of mIPSC amplitudes showed that this was likely due to the recruitment of additional GABA/glycinergic synaptic terminals, generating larger mIPSCs.

The effect of LP-211 on inhibitory transmission was blocked by 10 μM SB-269970, confirming the involvement of 5-HT_7_Rs (Figure 2F). Finally, the percentage frequency increase of sIPSCs resulted significantly higher than that observed for sEPSCs, indicating that 5-HT_7_Rs exert a more effective potentiation on inhibitory synaptic transmission (Figure 2G).

Since 5-HT_7_R expression has been detected on DRG neurons and primary afferent fibers, we have then tested whether LP-211 could modulate EPSCs evoked by dorsal root stimulation. A paired-pulse protocol was applied, recording two EPSCs at an interval of 400 ms; the stimulus intensity was 500 μM, able to recruit both Aδ and C fibers.

**FIGURE 3:**
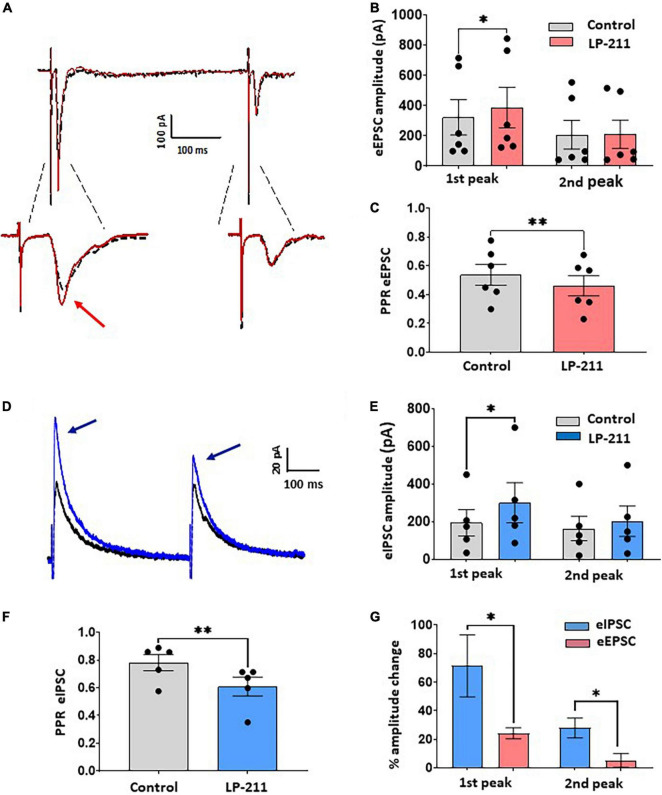
Application of LP-211 facilitates evoked excitatory postsynaptic currents (EPSCs) and Inhibitory postsynaptic currents (IPSCs), exerting a stronger action on inhibitory transmission. **(A)** Example of EPSC recordings (eEPSCs), evoked by dorsal root stimulation (500 mA, 0.1 ms) and recorded at –70 mV. Two stimuli were applied at 400 ms intervals (paired pulse protocol). LP-211 (1 μM, red trace) caused a moderate potentiation of evoked EPSCs in 5 out of 11 cells tested. **(B)** The first EPSC was significantly increased (Wilcoxon rank-sum test, *p* < 0.05, *n* = 6 EPSCs from 5 cells), while the second peak was not changed (Wilcoxon rank-test, *p* = 0.76, *n* = 6). **(C)** Paired pulse ratio (PPR = second EPSC/first EPSC) was significantly decreased by LP-211, suggesting a presynaptic action (paired *t*-test, *p* < 0.01, *n* = 6). **(D)** Recordings of IPSCs evoked by focal stimulation (paired pulse protocol) and recorded at 0 mV, in the presence of NBQX (10 μM) and D-AP5 (50 μM): in this example, LP-211 caused the increase of both IPSCs (blue trace). **(E)** The first IPSC underwent a stronger potentiation than the second peak, as observed in 5 responsive cells out of 11 (first peak, paired *t*-test, *p* < 0.05; second peak: paired *t*-test, *p* = 0.07, *n* = 5). **(F)** Paired pulse ratio was significantly decreased (paired *t*-test, *p* < 0.01, *n* = 5), confirming a presynaptic effect. **(G)** Evoked inhibitory currents were more affected by 5-HT_7_R activation compared to excitatory responses, as revealed by the higher percentage increase of both evoked IPSC peaks (Mann–Whitney test, *p* < 0.05, *n* = 5 and 6 for IPSCs and EPSCs, respectively). Asterisks reported in the graphs represent statistical significance: *p < 0.05; ^**^p < 0.01; ^***^p < 0.001.

As shown in Figures 3A–C, a significant increase in the first evoked EPSC is observed in 5 out of 11 lamina II neurons, which is not accompanied by a significant change in the second peak (first peak percentage change: 24.3 ± 3.7%; second peak: 5.3 ± 4.8%, Figure 3B). The paired-pulse ratio (PPR) of the evoked EPSCs was significantly decreased, suggesting a presynaptic site of action of 5-HT_7_Rs on primary afferent terminals (Figure 3C).

Evoked IPSCs, mediated by GABA and/or glycine, were strongly facilitated by LP-211 (Figures 3D–F). The IPSCs were elicited by focally stimulating lamina II with the paired-pulse protocol and were recorded in the presence of NBQX and D-AP5 to block glutamatergic transmission (Figure 3D). The activation of 5-HT_7_Rs by 1 μM LP-211 induced an increase of both IPSC peaks in 5 out of 11 tested neurons (first peak percentage change: 71.2 ± 21.6%; second peak: 27.9 ± 6.9%, Figure 3E). The stronger potentiation of the first IPSC, as compared to the second, resulted in a significant decrease in the PPR (Figure 3F). This is consistent with the results on spontaneous IPSCs, suggesting a presynaptic modulation exerted by 5-HT_7_Rs on inhibitory neurotransmitters. Similar to what was shown above for spontaneous synaptic transmission, a comparison between percentage changes of evoked EPSCs and IPSCs confirmed a stronger potentiation exerted by LP-211 on GABA/glycine mediated transmission (Figure 3G).

**FIGURE 4:**
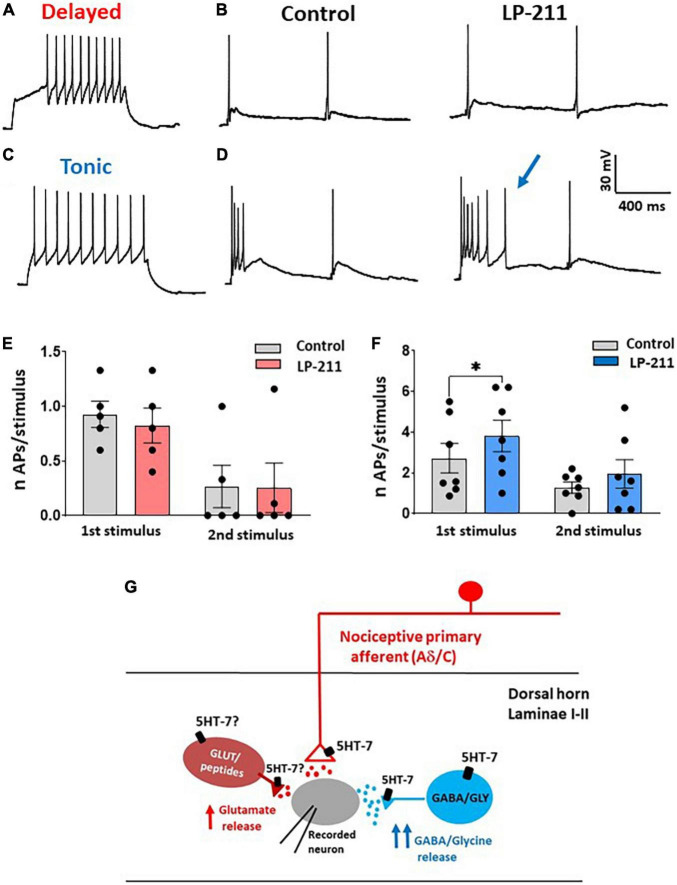
Activation of 5-HT_7_Rs differently affects the excitability of delayed and tonic firing lamina II neurons. **(A)** Example of a delayed firing neuron, recorded in current clamp at –80 mV. **(B)** In the same neuron, dorsal root stimulation (2 pulses) evoked action potentials (APs) superimposed to the excitatory postsynaptic potentials (EPSPs). The number of APs was not changed in the presence of LP-211 (1 μM). **(C)** Example of a tonic firing neuron, recorded at a potential of about –80 mV. **(D)** In the same neuron, LP-211 increased the number of APs elicited by root stimulation at the first pulse. **(E)** The mean number of APs evoked in delayed firing neurons was not changed by LP-211 (first stimulus: paired *t*-test, *p* = 0.08; second: paired *t*-test, *p* = 0.85, *n* = 5). **(F)** In the tonic firing neurons, the number of APs was significantly increased at the first stimulus (first: Wilcoxon rank-sum test, *p* < 0.05; second: paired t-test, *p* = 0.2, *n* = 7). **(G)** Schematic diagram depicting the hypothetical dorsal horn circuit involved in 5-HT_7_R-mediated synaptic modulation (see section “Discussion”). Asterisks reported in the graphs represent statistical significance: *p < 0.05; ^**^p < 0.01; ^***^p < 0.001.

Based on this consideration, we finally determined whether LP-211 can modify the excitability of lamina II inhibitory interneurons. As shown by previous studies, excitatory and inhibitory lamina II neurons exhibit different action potential firing patterns in response to current steps. While excitatory neurons show a delayed firing pattern, most inhibitory interneurons tend to fire tonically (Figures 4A,C). Based on this classification, we established the firing pattern of the recorded neuron at the beginning of the experiment. Afterward, we tested the effect of LP-211 on the number of action potentials evoked by dorsal root stimulation (paired-pulse protocol). The LP-211 did not affect the number of action potentials in delayed firing neurons (Figures 4B,E). Still, it effectively increased action potential firing at the first stimulus in tonic neurons (Figures 4D,F). The higher impact of 5-HT_7_R activation on the excitability of tonic firing neurons is consistent with the stronger effect exerted by LP-211 on inhibitory synaptic transmission.

## Discussion

This study has investigated the modulatory action exerted by 5-HT_7_Rs on dorsal horn synaptic transmission. Our results showed that 5-HT_7_Rs are able to potentiate both excitatory (mediated by glutamate) and inhibitory transmission (mediated by GABA and glycine), with a stronger impact on synaptic inhibition. The increase in frequency of spontaneous excitatory and inhibitory currents, not accompanied by changes in amplitude, and the decrease of PPRs in the evoked currents indicate that 5-HT_7_Rs act at presynaptic sites modulating neurotransmitter release.

In our experiments, we used the compound LP-211 to activate 5-HT_7_Rs. This agonist was tested previously in the hippocampus (Costa et al., 2012, 2015, 2018) and the cerebellum (Lippiello et al., 2016), but it was never utilized in studies on spinal cord neurons. In the hippocampus, modulatory effects of 5-HT_7_Rs have been described by applying very low concentrations of LP-211 (10 nM). We have observed reliable results only administering LP-211 at 1 μM, while 0.1 μM was ineffective. Similarly, LP-211 was tested at 1 μM on cerebellar slices, where 5-HT_7_Rs were involved in the induction of Long term depression (LTD) (Lippiello et al., 2016). The necessity of using higher concentrations of agonists in spinal cord slices as compared to other preparations could derive from their particular morphology and from the location of the recorded cells. Indeed, most recordings were obtained from neurons located quite deep into the slice, where synaptic connections are well preserved.

In our preparation, LP-211 was ineffective in potentiating excitatory transmission in the presence of the 5-HT_7_ antagonist SB-269970, while it produced significant effects when applied with the 5-HT_1*A*_ antagonist WAY-100635. SB-269970 was applied at the concentration of 10 μM that could also inhibit the activity 5-HT_5*A*_Rs. However, these receptors are negatively coupled to adenylate cyclase *via* Gi/o proteins whose activation opens K^+^ channels. This mechanism of action is not compatible with the increase of transmitter release and action potential firing observed in our study. Furthermore, SB-269970 administered at similar concentrations has proved to be ineffective on locomotor-like rhythmic activity recorded from *in vitro* spinal cord in 5-HT_7_R knockout mice (Liu et al., 2009). Interestingly, 5-HT_2_Rs that are involved in the generation of this activity together with 5-HT_7_Rs (Pearlstein et al., 2005) were not affected by SB-269970 at these concentrations.

Based on these considerations, we could assume that LP-211 selectively activates 5-HT_7_Rs in mouse spinal cord slices, even at higher concentrations. Future studies employing 5-HT_7_ knockout mice, shRNA, or CRISPR viral delivery will be helpful to further clarify the role of 5-HT_7_Rs in modulating nociceptive transmission in the spinal cord dorsal horn.

In the spinal cord dorsal horn, 5-HT_7_Rs have been observed on primary afferent fiber terminals (especially small myelinated and unmyelinated fibers), neuronal somas, dendrites, and astrocytes (Doly et al., 2005). Although 5-HT_7_Rs are expressed in both laminae I and II, we have focused our study on lamina II neurons, since a large number of studies are available to describe the correlation between action potential firing patterns and neuronal types in this region. This allowed us to determine the different effects of LP-211 on excitatory (delayed firing) and inhibitory interneurons (tonic firing, Figures 4A–F).

Dorsal horn neurons that express 5-HT_7_Rs are mainly represented by excitatory peptidergic neurons and GABAergic interneurons (Meuser et al., 2002; Doly et al., 2005). Consistently, we observed a 5-HT_7_-mediated potentiation of both spontaneous and evoked glutamate release. Furthermore, the effects of LP-211 on spontaneous and evoked GABA/glycinergic IPSCs confirm the role of 5-HT_7_Rs expressed on inhibitory interneurons. The diagram shown in Figure 4G summarizes our results in relation to a hypothetical dorsal horn circuit: functional 5-HT_7_Rs are expressed by some nociceptive afferent terminals synapsing onto the recorded lamina II neuron, by a subpopulation of inhibitory interneurons and probably also by some excitatory interneurons. Indeed, the effect of LP-211 on spontaneous EPSCs was reduced in the presence of TTX, suggesting that 5-HT_7_Rs expressed on excitatory interneurons could contribute to the modulation of glutamate release through an action potential-dependent mechanism.

Several cellular mechanisms could be involved in the synaptic facilitation mediated by 5-HT_7_Rs (Ciranna and Catania, 2014). As shown in many CNS areas, activation of these receptors can increase neuronal excitability by (i) reducing action potential hyperpolarization (through the inhibition of a Ca^2+^-dependent potassium current; Goaillard and Vincent, 2002); (ii) inhibiting the I_*A*_ potassium current (Siwiec et al., 2020); (iii) enhancing the hyperpolarization-activated cation current (Ih; Larkman and Kelly, 1997; Cardenas et al., 1999; Chapin and Andrade, 2001; Bickmeyer et al., 2002; Tang and Trussell, 2015); and (iv) potentiating the activity of voltage-dependent T-type calcium channels (Lenglet et al., 2002). Our study showed that LP-211 is particularly effective in increasing the excitability of tonic firing neurons, which mainly correspond to inhibitory interneurons (Daniele and MacDermott, 2009; Yasaka et al., 2010; Hughes et al., 2012). Interestingly, tonic-firing cells in lamina II have the highest expression level of Ih, which has also been often associated with inhibitory neuron populations (Hantman and Perl, 2005; Hughes et al., 2012; Smith et al., 2015). Together with the expression of T-type Ca^2+^ channels in some neuron populations (Smith et al., 2015), this property would make tonic firing inhibitory interneurons favorable targets for 5-HT_7_R-mediated modulation. The persistence of the LP-211 effect on mIPSCs in TTX would also suggest the presence of an action potential-independent, direct modulation at the inhibitory neuron axon terminal, similarly to what was observed in the hippocampus (Tokarski et al., 2011).

The effects of 5-HT_7_R activation on pain transmission *in vivo* are still under debate. Studies performed in rodents have reported both anti- and pro-nociceptive actions of these receptors. Analgesic effects seem to be prevalent for spinal 5-HT_7_R, while receptors expressed peripherally could be more involved in enhancing pain (Cortes-Altamirano et al., 2018). An anti-nociceptive action of 5-HT7Rs has been observed, for example, in models of pain hypersensitivity induced by capsaicin injection (Brenchat et al., 2009), sciatic nerve ligation (Brenchat et al., 2010), or chronic constriction injury (Viguier et al., 2012). Similarly, in mice, 5-HT_7_ agonists LP-211 and LP-44 induce analgesic effects on formalin-induced orofacial pain (Demirkaya et al., 2016). GABAergic interneurons seem to be especially involved in 5-HT_7_-mediated anti-nociception since the intrathecal administration of the GABA_*A*_ antagonist bicuculline prevented the anti-hyperalgesic effect of 5-HT_7_ agonists in rats with the constriction injury of the sciatic nerve (Viguier et al., 2012, 2013). On the other hand, pro-nociceptive actions of 5-HT_7_Rs have been described by Rocha-González et al. (2005), reporting an increase of flinching during the second phase of the formalin test after administration of a 5-HT_7_ agonist. In a different study, tactile allodynia induced by nerve injury was reduced by systemic or spinal administration of a 5-HT_7_ antagonist (Amaya-Castellanos et al., 2011). These discrepancies in 5-HT_7_R-mediated effects on animal models of pain *in vivo* could be explained by the differences in the pain models used and the low selectivity of some of the 5-HT_7_ agonists employed in the studies.

Our results, showing a prevalent effect of LP-211 in potentiating synaptic inhibition in naïve mice, suggest that 5-HT_7_Rs exert an anti-nociceptive effect in acute pain. However, it is difficult to predict how these modulatory mechanisms could be altered in conditions of chronic pain. As discussed by Bannister et al. (2017) in an interesting study demonstrating the involvement of 5-HT_7_Rs in spinal diffuse noxious inhibitory control (DNIC), the recruitment and function of dorsal horn 5-HT_7_Rs can be influenced by several factors, such as the pain state, the amount of serotonin release, the level of receptor expression in primary nociceptors and dorsal horn interneurons, and the relative contribution of other serotonergic receptors. Future experiments performed on identified dorsal horn neuron populations (excitatory or inhibitory interneurons and projection neurons) will allow a better characterization of 5-HT_7_R modulatory effects on dorsal horn neural circuits. These studies should be carried out in different animal pain models, determining the modifications of receptor expression and function in specific classes of neurons.

## Data Availability Statement

The raw data supporting the conclusions of this article will be made available by the authors, without undue reservation.

## Ethics Statement

The animal study was reviewed and approved by Italian Ministry of Health.

## Author Contributions

AC performed part of the experiments. EL and ML synthesized the compound LP-211 and wrote the manuscript. RB conceived and designed the study, performed part of the experiments, and wrote the manuscript. All authors contributed to the article and approved the submitted version.

## Conflict of Interest

The authors declare that the research was conducted in the absence of any commercial or financial relationships that could be construed as a potential conflict of interest.

## Publisher’s Note

All claims expressed in this article are solely those of the authors and do not necessarily represent those of their affiliated organizations, or those of the publisher, the editors and the reviewers. Any product that may be evaluated in this article, or claim that may be made by its manufacturer, is not guaranteed or endorsed by the publisher.
